# Functional Characterization of a Chimeric Soluble Fas Ligand Polymer with In Vivo Anti-Tumor Activity

**DOI:** 10.1371/journal.pone.0054000

**Published:** 2013-01-09

**Authors:** Sophie Daburon, Christel Devaud, Pierre Costet, Aurore Morello, Laure Garrigue-Antar, Mike Maillasson, Nathalie Hargous, Delphine Lapaillerie, Marc Bonneu, Julie Dechanet-Merville, Patrick Legembre, Myriam Capone, Jean-François Moreau, Jean-Luc Taupin

**Affiliations:** 1 Unité Mixte de Recherche Centre National de la Recherche Scientifique 5164, Université de Bordeaux 2, Bordeaux, France; 2 Animalerie spécialisée, Université de Bordeaux 2, Bordeaux, France; 3 Unité Mixte de Recherche Centre National de la Recherche Scientifique 7149, Université Paris-Est Créteil, Créteil, France; 4 Unité Mixte de Recherche Institut National de la Santé et de la Recherche Médicale 892, Université de Nantes, Nantes, France; 5 Centre génomique fonctionnelle, Université de Bordeaux 2, Bordeaux, France; 6 Equipe Associée 4427, Institut de Recherche en Santé-Environnement-Travail, Université de Rennes 1, Rennes, France; 7 Laboratoire d'Immunologie et immunogénétique, Centre Hospitalier et Universitaire de Bordeaux, Bordeaux, France; University of Birmingham, United Kingdom

## Abstract

Binding of ligand FasL to its receptor Fas triggers apoptosis via the caspase cascade. FasL itself is homotrimeric, and a productive apoptotic signal requires that FasL be oligomerized beyond the homotrimeric state. We generated a series of FasL chimeras by fusing FasL to domains of the Leukemia Inhibitory Factor receptor gp190 which confer homotypic oligomerization, and analyzed the capacity of these soluble chimeras to trigger cell death. We observed that the most efficient FasL chimera, called pFasL, was also the most polymeric, as it reached the size of a dodecamer. Using a cellular model, we investigated the structure-function relationships of the FasL/Fas interactions for our chimeras, and we demonstrated that the Fas-mediated apoptotic signal did not solely rely on ligand-mediated receptor aggregation, but also required a conformational adaptation of the Fas receptor. When injected into mice, pFasL did not trigger liver injury at a dose which displayed anti-tumor activity in a model of human tumor transplanted to immunodeficient animals, suggesting a potential therapeutic use. Therefore, the optimization of the FasL conformation has to be considered for the development of efficient FasL-derived anti-cancer drugs targeting Fas.

## Introduction

FasL (CD95L) is a type II homotrimeric transmembrane protein of the Tumor Necrosis Factor family of cytokines [1]. FasL is expressed on activated T lymphocytes and natural killer cells, as a weapon to eliminate transformed and infected cells expressing the transmembrane receptor Fas (CD95/APO-1) [2]. The triggering of Fas initially appeared as a promising approach to treat cancer but an agonistic anti-Fas antibody triggered fulminant lethal hepatitis upon injection in mice, precluding the use of Fas inducers for a therapeutical purpose in human [3].

Cleavage of membrane-bound FasL by a metalloprotease [4], [5] generates soluble homotrimeric FasL (sFasL), which is weakly apoptotic, and competes with membrane FasL for cell killing [6], [7]. Interestingly, upon cross-linking with antibodies, sFasL recovers its pro-apoptotic activity, and a FasL hexamer appears as the smallest functional form [8]. Similarly, agonistic anti-Fas monoclonal antibodies (mAbs) are mostly of the IgM or the self-aggregating IgG3 isotypes.

Our general aims were to develop new isoforms of functional FasL which do not require any crosslinking agent to become cytotoxic, to use them for deciphering the functional requirements leading to Fas activation, and to test them for in vivo anti-tumor activity. To reach the first goal, we fused the ectodomain of FasL to the modules of the extracellular domain of the LIF cytokine receptor gp190 [9] which display a propensity to self-associate [10], [11]. The gp190 belongs to the family of the hematopoietin receptors, characterized by the extracellular consensus Cytokine Binding Domain (CBD). The gp190 harbors two CBDs (D1 and D2) separated by an immunoglobulin-like (Ig) module. Therefore, the trimeric structure of the sFasL moiety, combined to the propensity of the gp190 modules to self-associate, could lead to differently aggregated sFasL chimeras with distinct apoptotic abilities.

To reach the second goal, we hypothesized that the distinct sizes of the gp190 modules (i.e. around 20, 40 and 100 kDa for Ig, D2 and D1IgD2 respectively), could exert different steric effects, distinctly impinging on the ability to trigger a productive apoptotic signal independently of the polymerization of FasL. In addition, given that Fas activation requires oligomers beyond the trimeric stage, we reasoned that either aggregation of the trimers, or a particular conformational change within a single trimer triggered by a polymeric ligand, or both, is mandatory. Therefore, we wondered whether anti-Fas antibody, naturally occurring sFasL and our chimeras, would be able to stimulate a chimeric Fas receptor which would only require dimerization to transmit a signal, and whether or not this property would correlate with the ability to trigger cell apoptosis. To explore this possibility, we used the gp130 signal transducing cytokine receptor, another member of the hematopoietin receptors, which is pre-assembled as dimers [12] and requires a ligand-induced conformational change to become activated. Gp130 triggers cytokine-dependent proliferation of various cell lines via the Jak-STAT pathway [13]. We fused transmembrane and intracellular regions of gp130 to the extracellular region of Fas, generating the Fas-gp130 receptor, and expressed it in the BA/F3 cell line.

To reach our third goal, in vivo toxicity in normal mouse, and ability to counteract tumor development in a model of human solid tumor transplanted into immunodeficient mice were explored for our most efficient sFasL chimera.

## Materials and Methods

### Antibodies and reagents

Anti-FasL mAb 14C2 and 10F2 used for the FasL ELISA [14], IgG anti-human Fas mAb 5D7 [14], isotype-matched negative controls 1F10 (IgG) and 10C9 (IgM) mAbs [15] were all generated in the laboratory. Chimeric Fas-Fc receptor was produced in the laboratory and was affinity-purified on protein A. Anti-FasL mAb (G247) used for immunoblots and anti-human Fas non agonistic mAb DX2 were purchased from BD Biosciences (Le-Pont-De-Claix, France). Recombinant sFasL (recFasL) was purchased from Alexis Corporation (Coger, Paris, France), and used with its cross-linking “enhancer” reagent, as recommended by the manufacturer. Anti-human Fas agonistic mAb 7C11 (IgM) was from Immunotech (Marseille, France). Anti-murine Fas agonistic mAb (JO2) was from Bender MedSystems (Vienna, Austria).

### Construction of the FasL chimeras

The isolation of the gp190 receptor modules Ig, D2 and D1IgD2 (amino acids 246 to 328, 329 to 542 and 49 to 542), was described previously [10]. They were fused to the extracellular domain of hFasL (amino acids 108 to 281) isolated by PCR. To avoid confusion with the immunoglobulin Fc fragment, the IgFasL chimera is called pFasL for polymeric soluble FasL. To generate the Fas-gp130 chimera, the Fas extracellular region and the transmembrane and intracellular domains of gp130 were isolated by site-directed mutagenesis and fused together. All the constructs were subcloned into the pED4 eukaryotic expression vector [16].

### Cell lines and transfections

The cells were grown in a 5% CO2 incubator at 37°C without antibiotics in medium supplemented with 8% FCS (Sigma, Saint-Quentin-Fallavier, France). Culture medium was RPMI for the human Jurkat T-lymphoma and the BA/F3 pro-B-lymphocytic murine cell lines, and DMEM for the human skin carcinoma A431 and the simian epithelial COS cell lines.

COS cells were transiently transfected using the DEAE-dextran method, with 5 μg of plasmid DNA, and supernatants were harvested 5 days later. Large scale production of pFasL was performed in serum-free Opti-MEM medium (Fischer Scientific, Illkirch, France).

The BA/F3 culture medium was supplemented with 10% WEHI cell-conditioned medium as a source of murine interleukin (IL)-3. BA/F3 cells (5.10^6^ cells in 0.3 ml) were electroporated (BTM 830 electroporator, BTX Instruments, Holliston, MA). G418 at 1 μg/ml (Invitrogen) was added at day 1. The G418-resistant cells were cloned by limiting dilution in the presence of murine IL-3. Stable transfectants were selected for membrane expression of the Fas-gp130 molecule by flow cytometry with the anti-Fas antibody 5D7. BA/F3 cells were washed three times to remove IL-3, then incubated with the indicated ligands, and proliferation was estimated using the MTT proliferation assay after 3 days, as described previously [10]. The maximum value and the blank value were obtained with a saturating concentration of IL-3 or without IL3, respectively.

The BA/F3, Jurkat, COS and A431 cell lines were obtained respectively in 1991, 1995, 1992 and 2004 from Drs D'Andrea [17], Anderson [18], Kaufman [19] and Nagata [20]. They were mycoplasma-tested every 6 months by PCR [21] and Hoechst 33258 staining [22]. Absence of cross-contamination was verified almost daily by morphology check for all the cell lines, and by growth curve analysis in the presence and absence of IL-3 for the BA/F3 cell line.

### ELISA for sFasL

FasL was quantified in cell culture supernatants using a conformation-dependent home made sandwich ELISA based on non-blocking mAb 14C2 (10 μg/ml) as a capture antibody and blocking biotinylated mAb 10F2 (1 μg/ml) as a tracer. All steps were performed exactly as reported for our anti-human LIF ELISA [23].

### Western blot analysis

Supernatants from transfected cells were harvested and debris were removed by centrifugation. FasL was quantified and 100 ng of the FasL protein were resuspended in 5x Laemmli buffer and separated by SDS-PAGE on 12% gels. Proteins were transferred to a polyvinyldifluoride membrane (Amersham, Buckinghamshire, England) and immunoblots were performed as previously described [24]. The anti-FasL mAb G247 (1 μg/ml) was incubated overnight at 4°C. BN-PAGE was carried out as described by Schägger [25] with the following modifications. A separating 4–18% w/v acrylamide linear gradient was used. Before loading, 1 μL of sample buffer (500 mM 6-amino-*n*-caproic acid, 5% w/v Serva Blue G) was added to the sample. The gel was run overnight at 4°C with 1 W. Thyroglobulin (669 kDa) and BSA (66 kDa) were used as size standards (Sigma). FasL was detected using mAb G247.

### Surface plasmon resonance analysis of the FasL chimeras binding to Fas

The experiments were carried out on a BIAcore 3000 optical biosensor (GE healthcare, Chalfont, UK). The FasL chimeras were produced as COS supernatants in Opti-MEM medium, concentrated 100 times, dialyzed against PBS and sterilized by filtration. Recombinant Fas-Fc (R&Dsystems, Minneapolis, MN) was covalently coupled to a carboxymethyl dextran flow cell (CM5, BIAcore) following the manufacturer's recommendations. The level of immobilization was 2,000 resonance units (RU). Binding of the FasL chimeras was assayed at concentrations ranging from 0.2 to 100 nM for IgFasL, 0.2 to 44 nM for cFasL, 0.2 to 37.5 nM for D2FasL, and 0.25 to 8 nM for D1IgD2FasL, in Hepes-buffered saline, at a 30 **μ**l/min flow rate. Association was monitored for 5 min before initiating the dissociation phase for another 11 min with Hepes-buffered saline. The flow cell was regenerated with 4M MgCl2. The sensorgrams were analyzed using the BIAeval 4.1 software (BIAcore). The background of the Opti-MEM medium was at 30 RU.

### Cell cytotoxicity assays

The cytotoxic activity of the FasL chimeras was measured using the MTT viability assay as previously described, after 24 hours of incubation with serial dilutions of the indicated ligands [14]. The percent of specific cytotoxic activity of FasL was calculated as follows: 100 – (experimental absorbance – background absorbance)/(control absorbance – background absorbance) ×100.

### Gel filtration experiments

The molecular size of the FasL constructs was determined using the size exclusion S-200-HR and S-300-HR Sephacryl columns of 120 ml bed volume and 40 ml void volume (Amersham Pharmacia, Orsay, France). COS supernatants were concentrated with Centricon-30 (Millipore, Saint-Quentin-en-Yvelines, France) to reach 2 μg/ml for each sFasL form. One microgram was loaded onto a column and eluted in PBS at 0.3 ml/min. 80 Fractions of 0.8 ml were collected starting at 25–30 ml of elution, and were analyzed for the presence of FasL protein by ELISA and for Fas-mediated cytotoxicity using the MTT assay.

### FasL purification and mice injection

Experiments with normal Balb/cByJCr1 mice used immunoaffinity purified pFasL. Supernatant from transfected COS cells (500 mL) was immunoprecipitated using 1 ml of anti-FasL mAb (14C2)-coupled NHS-activated sepharose beads (Amersham), overnight at 4°C. Beads were pelleted and washed in PBS, and pFasL was eluted at pH 2 (50 mM glycine, 1 M NaCl). The eluate was immediately neutralized by adding 0.25 volume of 1 M Tris–HCl buffer at pH 8. After overnight dialysis against PBS, FasL was quantified by ELISA. Male BALB/cByJCr1 mice (8 wk old) were injected intraperitoneally with 0.5 ml PBS containing 10 μg of pFasL, or of anti-Fas agonistic mAb JO2, or with PBS alone. Blood was collected at 6 and 30 h for liver enzymes measurement. The mice were euthanasied at 30 h post-injection.

For tumor experiments, COS cells were transfected with pFasL or empty vector as a control, and grown in Opti-MEM medium. Supernatants were harvested at day 5, centrifuged, concentrated 60 times against polyethylene glycol flakes, adjusted to 0.1 mg/ml and sterilized by filtration. Immunodeficient Rag^−/−^γc^−/−^ mice, a gift from Dr Di Santo [26], were used at 7–10 weeks of age, and housed in appropriate animal facility under pathogen-free conditions. At day 0, mice received 10^5^ A431 cells in 0.1 ml of culture medium subcutaneously into the right flank. Injections of pFasL (10 μg in 0.1 ml) or concentrated empty vector control supernatant were performed after tumor implantation, either subcutaneously at days 2 and 7, or intraperitoneally everyday between days 0 and 7, then at days 9, 11 and 14. Tumor growth was monitored by measuring maximal and minimal diameters with a calliper, three times a week, and tumor volume was estimated with the formula: tumor volume (mm^3^) =  length (mm) × width^2^ (mm) ×0.5. The animal studies conducted in this report have been reviewed and approved by the scientific committee from the Bordeaux Segalen University's animal facility. Experimental work was performed by Dr Capone with agreement reference C33 00 024. All efforts were made to minimize suffering. Mice were euthanized at day 35, as no therapeutic effect of IgFasL or control were expected any more at that point of tumor evolution with the experimental design used. At this time point, tumor volume remained below 1500 mm^3^.

### Statistical analysis of tumor growth

The Mann-Whitney test was used for the comparison between the two groups in the experiment with subcutaneous injection of pFasL. The Kaplan-Meier analysis was used to establish the survival curves without cancer, and comparison between the two groups was made using the log-rank test. Analyses were performed with Statview Software (Abacus Concepts, Berkeley, CA). For all experiments, a p≤0.05 was considered significant.

## Results

### Generation and production of soluble potentially multimeric FasL/gp190 chimeras

**Figure 1:**
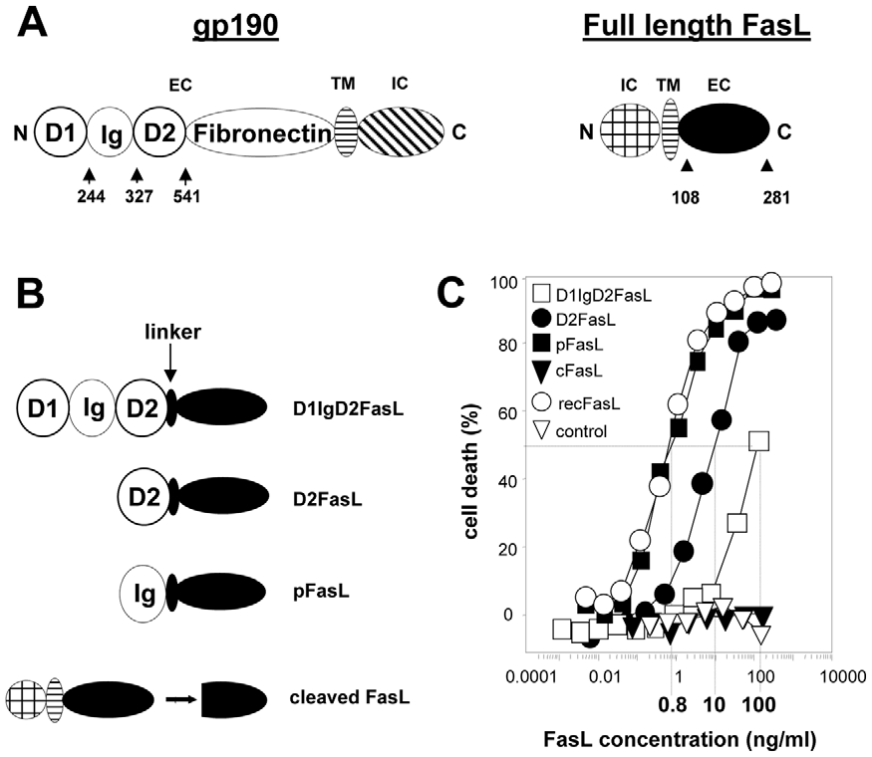
Obtention and functional characteristics of the FasL/gp190 chimeras. **Panel A:** Modules constituting gp190 and FasL are depicted as mature proteins. EC, TM and IC represent the extracellular, transmembrane and intracellular domains, respectively. N and C represent the N- and C-terminal ends. The numbers depict the domain boundaries used to create the chimeras. Cleaved FasL (cFasL) is spontaneously generated by a metalloprotease cleaving between aminoacids 126 and 127. **Panel B:** Representation of the cFasL and the gp190/FasL chimeras. **Panel C:** Serial dilutions of supernatants from COS cells transfected with the FasL constructs or the empty vector (control) were incubated with Jurkat cells. Cell death was measured using the MTT assay. As a positive control, we used the commercially available antibody-cross-linked FasL (recFasL). Calculated C50 are indicated on the graph. Results from one representative experiment out of 5 are depicted.

We fused the Ig-like, D2 and D1IgD2 modules of gp190 to the FasL extracellular region (Figures 1A and 1B). The constructs were expressed in COS cells and the secreted molecules were quantified using a FasL-specific ELISA. To measure their ability to trigger cell death, we incubated serial dilutions of the supernatants from chimeric FasL, control mock-transfected and wild-type FasL transfected cells with Fas-sensitive Jurkat cells. A commercially available FasL (recFasL) was also tested as a highly active reference. The pFasL was the strongest death inducer among our chimeras and was as powerful as recFasL (Figure 1C). D2FasL and D1IgD2FasL were respectively 12.5 and 125 times less potent than pFasL. As already known, spontaneously cleaved membrane FasL (cFasL) had almost no activity [6], [7]. The concentration of the anti-Fas agonistic IgM antibody 7C11 required to kill 50% of the Jurkat cells was at 2 ng/ml (results not shown) [14], [24], [27].

### Biochemical characterization of the FasL/gp190 chimeras

**Figure 2:**
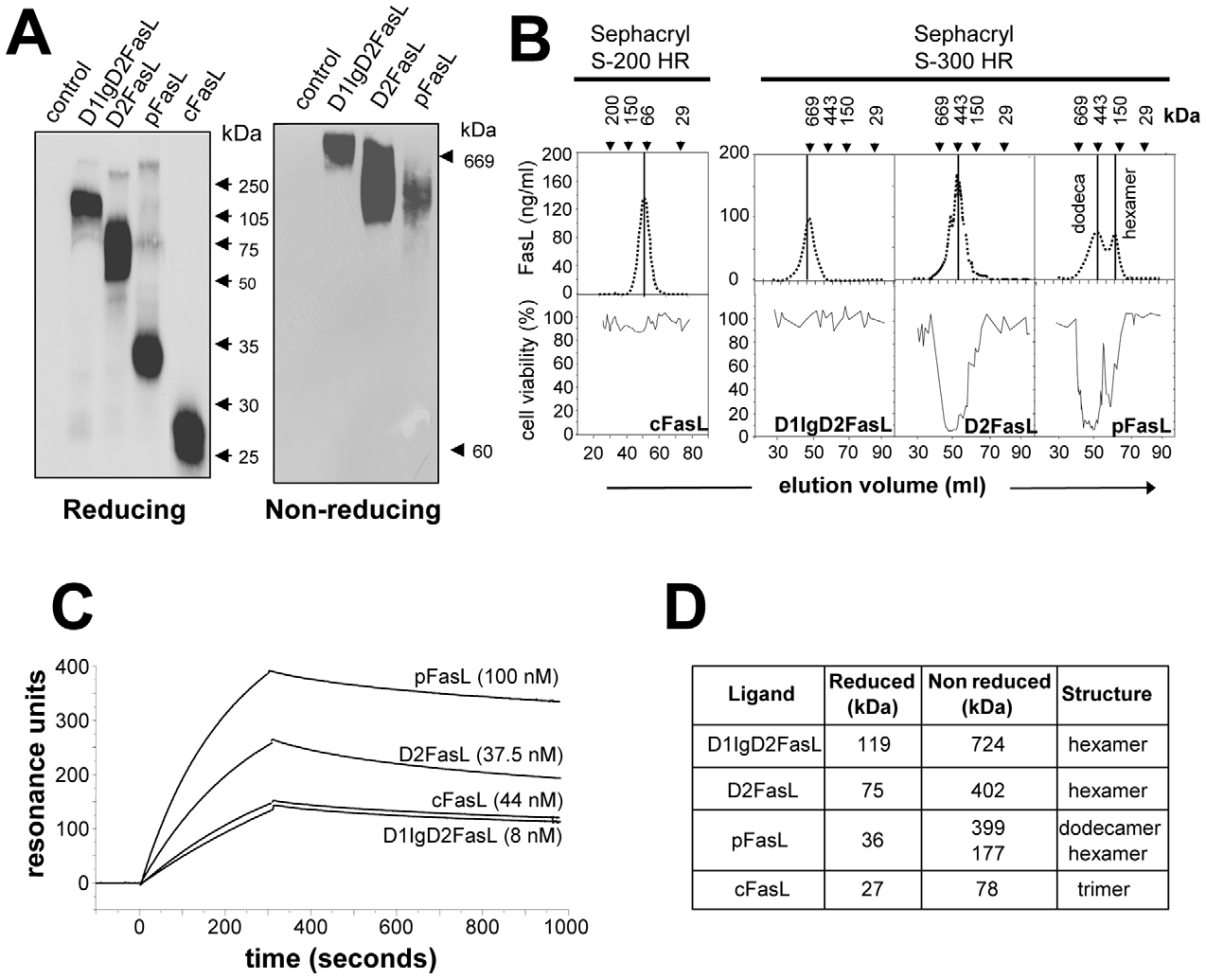
Biochemical characterization of the FasL/gp190 chimeras. **Panel A:** Supernatants from COS cells transfected with the FasL constructs were quantified by ELISA and 10 μg of FasL protein were loaded per lane. Migrations were performed under reducing (SDS-PAGE) or non-reducing (BN-PAGE) conditions. FasL was revealed by immunoblot. **Panel B:** 2 μg of FasL construct were loaded on the gel filtration column. FasL was quantified by ELISA in elution fractions, and cytotoxicity was measured using the MTT assay. **Panel C:** Affinity measurement using Biacore®. Fas-Fc was immobilized on the chip, before the indicated soluble FasL constructs were added. A range of concentrations was tested for each analyte, but only the graph obtained with the highest concentration tested is displayed. **Panel D:** The apparent molecular weights and degree of oligo/polymerization of the FasL chimeras were estimated from the non denaturing gel electrophoresis and gel filtration experiments.

Identical amounts of the ^35^S-labeled FasL constructs were separated by SDS-PAGE (Figure 2A, left panel) and the molecular mass of each chimera was determined under reducing conditions (Figure 2D). We also performed native gel electrophoresis (BN-PAGE) in non-reducing conditions (Figure 2A, right panel), and observed that pFasL, D2FasL and D1IgD2FasL all displayed much higher molecular weights than expected from the SDS-PAGE analysis (Figure 2D). The three chimeras were also analyzed by gel filtration chromatography (Figure 2B). Elution fractions were analyzed for the presence of FasL by ELISA (Figure 2B, upper panel) and for cytotoxic activity against the Jurkat cell line (Figure 2B, lower panel). We confirmed that the metalloprotease-cleaved FasL is a non cytotoxic homotrimer, whereas D2FasL and D1IgD2FasL behaved as hexamers. In contrast to D2FasL, D1IgD2FasL was very weakly cytotoxic, in agreement with Figure 1C. The pFasL existed under two distinct forms corresponding to a high molecular weight dodecamer and to a smaller hexameric form. Both were cytotoxic, which is consistent with previously published results for soluble FasL in the case of the hexamer [8].

The affinity of the FasL chimeras for Fas was measured using the surface plasmon resonance Biacore® method, against recombinant Fas-Fc immobilized on a chip. D2FasL, D1IgD2FasL pFasL and sFasL as a control, were produced as supernatants in COS cells cultured in serum-free medium, concentrated 100 times, and dialysed against PBS. The sensorgrams are depicted in Figure 2C and the association and dissociation constants are presented in Table 1. The Kd for the three chimeras were very close to each other, ranging from 11.6 nM for pFasL, to 25.6 nM for D1IgD2FasL and to 38.5 nM for D2FasL. They were inversely correlated with the degree of polymerisation of the construct, and two to six times higher than for non-chimeric cFasL (Kd  = 69.4 nM). Therefore, the small differences between the chimeras did not significantly alter the ability of the FasL moiety to bind to Fas, nor did it explain the strong discrepancies in their abilities to trigger apoptosis.

**Table 1:** Association/dissociation constants of the soluble FasL chimeras.

Ligand	Kon (1/Ms)	Koff (1/s)	KD (M)	Chi^2^
D1IgD2FasL	1.3×10^5^	3.3×10^−3^	2.56×10^−8^	8.34
D2FasL	1.6×10^5^	6.0×10^−3^	3.85×10^−8^	3.37
pFasL	2.5×10^4^	4.1×10^−4^	1.16×10^−8^	2.75
cFasL	8.4×10^4^	5.9×10^−3^	6.94×10^−8^	16

### FasL chimeras and agonistic antibody differentially act on Fas conformation

**Figure 3:**
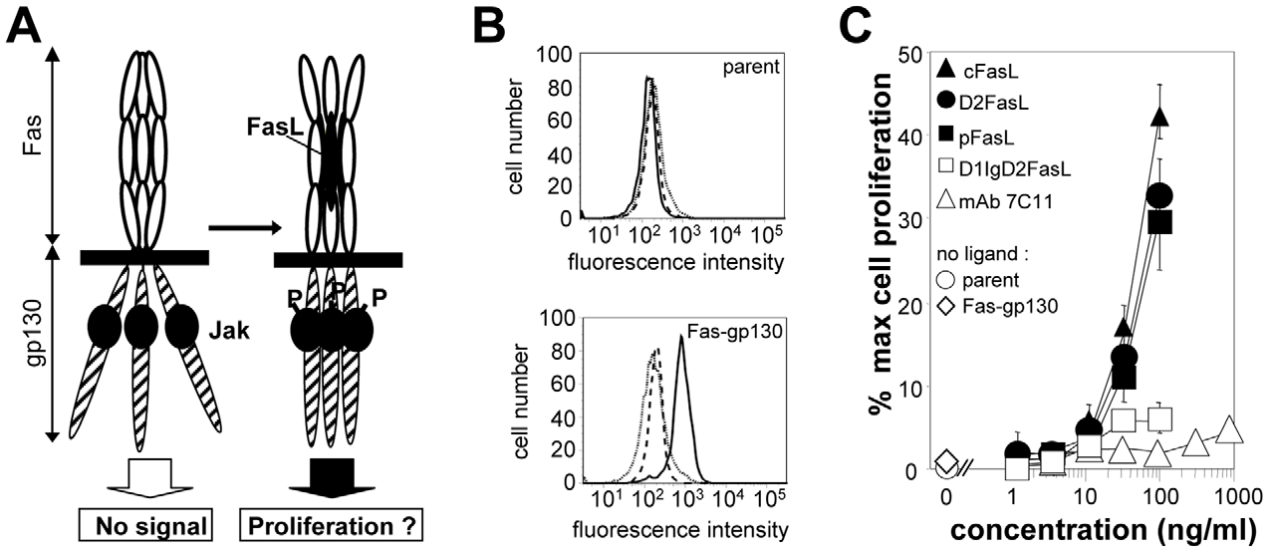
FasL/gp190 chimeras and agonistic antibodies differentially act on Fas conformation. **Panel A:** Description of the model used to analyze the requirement for a Fas conformational change during its activation. The Fas-gp130 hybrid receptor is stably expressed in the IL-3 dependent BA/F3 cell line. **Panel B:** Cell surface staining of parent BA/F3 cells (upper panel) and on a representative clone stably expressing the Fas-gp130 chimera (lower panel), with an isotype-matched control (dotted line), anti-murine Fas JO2 (dashed line) and anti-human Fas DX2 (continuous line). Living cells were gated on the basis of the morphological parameters. **Panel C:** Fas-gp130 BA/F3 cells were incubated with the indicated Fas triggers or controls, and proliferation was measured using a MTT assay. Results are expressed as percentages of the maximum proliferation obtained with a saturating IL-3 concentration. Proliferation of parent and transfected cells was also measured in the absence of any IL-3 or Fas trigger. Values are the mean ± sd of 3 independent experiments.

To determine whether a conformational change in the Fas receptor is required to produce the apoptotic signal, we generated a fusion protein between the extracellular region of Fas and the transmembrane and intracellular region of the gp130 hematopoietin receptor (Figure 3A) which we expressed in the IL-3 dependent BA/F3 murine cell line. This cell line relies on exogenously added cytokines to survive and proliferate, and also lacks membrane expression of murine Fas as shown by flow cytometry staining with the JO2 antibody (Figure 3B, upper panel). In the presence of FasL, stable expression of the chimera was expected to keep the cells proliferating through the activation of the gp130 pathway. The membrane expression of the Fas-gp130 chimera was verified using flow cytometry, and the absence of murine Fas on the transfectants was confirmed (see Figure 3B, lower panel, for the representative clone used in the proliferation experiments). In the absence of IL-3, the BA/F3 Fas-gp130 cells did not proliferate, demonstrating that the Fas-gp130 chimera by itself was not able to sustain cell growth (Figure 3C).

We then analyzed the effect on cell survival and proliferation of serial dilutions of the 7C11 agonistic anti-Fas antibody, of the FasL chimeras, and of cFasL (Figure 3C). Cell viability was expressed as the percentage of the maximal proliferation triggered by a saturating concentration of IL-3. We observed that the strongly apoptotic 7C11 mAb was not able to sustain cell proliferation. In contrast, the pro-apoptotic pFasL and D2FasL triggered a strong and quantitatively comparable proliferative signal, although D2FasL was 12.5 times less efficient than pFasL for killing the Jurkat cells (see Figure 1C). D1IgD2-FasL, which is hexameric like D2FasL but only weakly triggers cell death (see Figure 1C), was unable to sustain cell proliferation. The non apoptotic homotrimer cFasL which is unable to aggregate the pre-associated Fas homotrimers and as such is not cytotoxic, nevertheless triggered a proliferative signal comparable to that of D2FasL and IgD2FasL. The discrepancy between the polymeric apoptotic antibody 7C11 and the trimeric cFasL demonstrated that the proliferative signal did not require Fas aggregation, and suggested that the triggering of Fas may also include a ligand-induced conformational change of the receptor itself.

### Anti-tumor activity of pFasL

The pFasL chimera exerted its cytotoxic activity against various human tumor cell lines from distinct origins, both hematopoietic (CEM and H9 T-lymphoma cells, SKW6.4 and JY B-lymphoma cells, with C50 ranging from 0.01 to 0.1 μg/ml), and non-hematopoietic (A431 epidermoid carcinoma cells, which was weakly sensitive, with C50 = 0.15 μg/ml) (results not shown). We observed for these cell lines differences in efficiency of the apoptotic activity that were similar to pFasL, with the different chimeras (results not shown), demonstrating that the distinct behaviors of these chimeric proteins were independent of the cell type.

To determine the hepatotoxicity of pFasL, we injected the ligand in mice and we analyzed in peripheral blood the markers of liver injury aspartate amino transferase (ASAT) and alanine amino transferase (ALAT). Mice were injected intraperitoneally with 10 μg (0.7 μg/g) of affinity-purified pFasL diluted in PBS. As controls, one mouse was injected with an identical volume of PBS and another one was left untreated. As a positive control, two mice were injected intraperitoneally with 10 μg of the agonistic anti-murine Fas antibody JO2 in the same volume of PBS. One of these mice developed a fulminant hepatitis and was sacrificed 6 hours after antibody injection. The anti-Fas JO2 mAb triggered a rapid and considerable increase of both serum amino transferases, whereas sera from the negative control mice and mice injected with the purified pFasL did not show any sign of liver cytolysis (Table 2).

**Table 2:** pFasL does not induce liver damage.

Fas trigger	ASAT (IU/ml)		ALAT (IU/ml)	
	6 hours	30 hours	6 hours	30 hours
Control (no PBS)	66	48	43	27
Control (PBS)	84	61	49	58
Anti-Fas (JO2) (mice 1,2)	12 383, 1 419	ND^1^, 1 650	876, 27	ND^2^, 6 197
pFasL (mice 1, 2 and 3)	81, 80, 69	63, 205, 82	55, 31, 96	37, 50, 58

1 mice were injected with the indicated ligands as described in Materials and Methods. Blood samples were harvested at the indicated time points and the levels of alanine amino transferase (ALAT) and aspartate amino transferase (ASAT) were measured in the serum.

2 not determined, euthanised before reaching time point.

**Figure 4:**
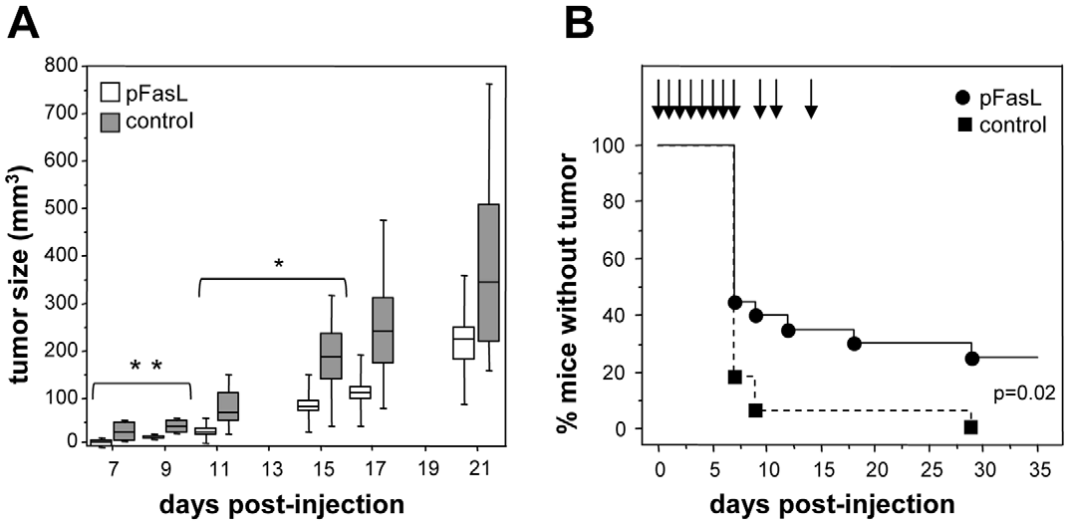
Anti-tumor activity of pFasL. **Panel A:** Tumor growth in mice having received subcutaneously 10^5^ A431 cells at day 0, and 0.1 mL of concentrated pFasL (white boxes) or pFasL-free control (grey boxes) locally at days 2 and 7 (n = 6 mice per group). Tumor volumes are expressed in mm^3^. Values are presented as median, 25^th^ and 75^th^ percentiles (horizontal line, bottom and top of boxes), and 10^th^ and 90^th^ percentiles (bottom and top range bars) (**p = 0.04, * p = 0.05). **Panel B:** Kaplan-Meier analysis of cumulative percentage of mice without detectable tumor,, xenografted with A431 cells and treated with pFasL (black circles) or pFasL-free control (black squares) (p = 0.02). n = 20 mice per group, from two experiments pooled.

The anti-tumor activity of pFasL was estimated in a mouse model, using the weakly Fas-sensitive human A431 cells transplanted subcutaneously to Rag^−/−^ γc^−/−^ immunodeficient mice. In a first experiment (Figure 4A), we analyzed whether pFasL injected locally would control tumor growth. For that, 10^5^ A431 cells were injected to two groups of 6 mice. Then the mice received two local subcutaneous injections of either pFasL (a non toxic amount of 10 μg in the form of a serum-free concentrated supernatant) or pFasL-free control, at days 2 and 7 after tumor implantation. Tumor growth was regularly measured until day 21, and the evolution of tumor volumes is depicted in Figure 4A. The local administration of pFasL significantly reduced tumor growth, in comparison to the mice injected with the control without pFasL, but the effect vanished when the injections were stopped. We next analyzed whether injection of pFasL at a distance from the tumor site would have a similar effect. For that, 10^5^ A431 cells were injected to two groups of 10 mice, and two independent experiments were performed. The mice received intraperitoneal injections of either pFasL (10 μg) or pFasL-free control, everyday from day 0 to day 7, and thereafter at days 9, 11 and 14 only. Tumor size was measured regularly until day 35. The percentage of mice without detectable tumor is presented in Figure 4B, and shows that pFasL is able to significantly (p = 0.02) lower tumor growth, as 25% of the mice having received pFasL remain tumor-free at a time where the control mice having received medium alone all displayed tumor development. Therefore, these in vivo preliminary experiments demonstrate that the in vitro biological properties of pFasL are conserved in vivo.

## Discussion

The pFasL, D2FasL and D1IgD2FasL chimeras allowed us to analyze the structure-function relationships enabling FasL to activate Fas. The cytotoxic activity strongly depended on both the polymerization level of the chimera and the size of its constitutive monomers, more than on the affinity for Fas, which was very close for all three. Indeed, the most efficient construct was pFasL, i.e. the most polymeric (dodecameric) but also the shortest one at the monomeric level. However, it is noteworthy that hexameric D1IgD2FasL was 10 times less cytotoxic than hexameric D2FasL, suggesting that the polymerization degree is not the only parameter to be important. In line with this, the IgM agonistic antibody 7C11 displays ten potential binding sites for Fas, and therefore should behave closely to the dodecameric pFasL. However, we previously demonstrated that pFasL can trigger apoptosis in cells harboring a mutation in the Fas death domain at the hemizygous state, which were completely insensitive to the agonistic antibody [24]. Therefore, our results confirmed that the extent of FasL oligomerization is essential but not sufficient for triggering the apoptotic signal. We therefore hypothesized that a Fas conformational change might be required as well.

We explored this possibility with the cellular assay using the Fas-gp130 chimeric receptor. Trimeric cFasL, pFasL and hexameric D2FasL efficiently triggered proliferation, but hexameric D1IgD2-FasL did not. It is possible that the voluminous D1IgD2 domain impairs the conformational change in the gp130 domain while maintaining Fas binding. This could similarly explain why it lacks cytotoxicity towards wild-type Fas. The agonistic anti-Fas antibody is also unable to trigger cell proliferation through Fas-gp130, although it efficiently triggers apoptosis [14], [27]. As for D1IgD2FasL, this could be explained by structural constraints due to the IgM isotype. The apoptotic effect of the IgM mAb would then result from a large aggregation of Fas trimers, leading to caspase activation. In line with this, the non apoptotic cFasL is expected to trigger strong cell proliferation, because it is the Fas natural ligand and as such must display the best fit for this receptor. As pFasL is capable of triggering the adequate Fas conformational change and is also polymeric, this would therefore explain why it can kill cells which normally resist to the agonistic antibodies [24]. These results overall confirm our reported finding that FasL and antibodies do not stimulate identically the Fas signalling machinery [27], and confirm the requirement of minimal Fas aggregation by a multimeric ligand trigger [8]. In addition, a recent work brought into the landscape the theory of the T-cell receptor serial triggering and applied it to Fas, by clearly demonstrating that there is an inverse correlation between agonistic antibody affinity and potency [28]. According to these authors, the antibodies with the highest affinity for Fas do not efficiently trigger apoptosis by remaining blocked on the receptors they bound to, precluding the formation of higher order receptor aggregates able to transmit the apoptotic signal. Our findings on the FasL chimeras are not contradictory to these results, although we observed drastic differences that could not be explained by affinity differences for Fas. On the contrary, our results strengthen the notion that Fas agonistic antibodies and FasL-derived agonists display intrinsic major peculiarities in their respective modes of action, which have to be considered for a therapeutic application in human.

The pFasL chimera demonstrated a very potent apoptotic activity, in the absence of any cross-linking enhancing agent. Using experiments in mouse, we detected no liver damage after intravenous injection. Although these findings seem in contradiction with data showing that Fas engagement in mice induce an acute liver injury, it is noteworthy that these reports used in fact the anti-Fas JO2 agonistic antibody and not FasL [3], [29], [30], [31], [32]. The liver destruction observed following injection of anti-Fas antibodies may simply be the consequence of an antibody-dependent cell-mediated cytotoxicity reaction [33], as the production of inflammatory cytokines by Fc receptor–bearing Kupffer cells has been observed [34]. In addition, our results confirm another report, which showed that injection of a polymeric leucine-zipper chimeric FasL in rats only triggered a mild liver damage [35]. Therefore, we predict that all forms of polymeric FasL which would depend on antibody-mediated cross-linking will be toxic. Using a transplanted human tumor mouse model, we then demonstrated an anti-tumor effect of a non-toxic dose of pFasL, administered several times, locally or intraperitoneally at a distance from the tumor site. Therefore, pFasL also demonstrated in vivo activity, by reducing tumor development. Interestingly, the A431 human epidermoid carcinoma cell line we used naturally displays only weak sensitivity to FasL-induced apoptosis when compared to the human lymphoid cell lines we tested. Although more experiments and higher doses are still required to better describe pFasL toxicity and activity, it appears that for a future therapeutic use in cancer treatment, the design of soluble FasL forms spontaneously reaching a high degree of polymerization should also consider their ability to trigger the adequate Fas receptor conformational adaptation.
